# Dirhamnolipids from *Pseudomonas aeruginosa* PAO1 Protect Hairless Mouse Skin from UVB-Induced Inflammation and Oxidative Stress

**DOI:** 10.1021/acsomega.5c09252

**Published:** 2026-06-19

**Authors:** Isadora Caroline Sawoniuk, Ingrid Caroline Pinto, Priscila Saito, Jonathan Ratko, Kamila B. B. Wessel, Renata M. Martinez, Cesar A. Tischer, Ricardo Luís Nascimento de Matos, Thaísa Maria da Roda Lino, Nicole Caldas Pan, Laura de Oliveira Semeão, Ana Paula Frederico R. L. Bracarense, Waldiceu A. Verri, Rubia Casagrande, Josiane A. Vignoli, Doumit Camilios-Neto

**Affiliations:** † Departamento de Bioquímica e Biotecnologia, Centro de Ciências Exatas, 37894 Universidade Estadual de Londrina, 86057-970 Londrina, Brazil; ‡ Departamento de Ciências Farmacêuticas, Centro de Ciências da Saúde, 37894 Universidade Estadual de Londrina, 86038-440 Londrina, Brazil; § Departamento de Medicina Veterinária, Centro de Ciências Agrárias, 37894 Universidade Estadual de Londrina, 86057-970 Londrina, Brazil; ∥ Departamento de Imunologia, Parasitologia e Patologia Geral, Centro de Ciências Biológicas, 37894 Universidade Estadual de Londrina, 86057-970 Londrina, Brazil

## Abstract

Frequent and excessive skin exposure to ultraviolet radiation induces oxidative stress and inflammation, raising skin diseases. Therapeutic strategies targeting both oxidative stress and inflammation may reduce skin disorders. Rhamnolipids are highly effective surface-active glycolipid biosurfactants with enormous market potential. Although these green amphiphilic molecules exhibit remarkable biomedical potential, including antimicrobial activity, wound healing properties, and analgesic effect on inflammatory pain, their therapeutic potential in UVB induced skin inflammation has not yet been investigated. A glycolipid product enriched in Dirhamnolipids (95.7%) was obtained, followed by single-step purification that provided a highly pure fraction containing 99.5% total rhamnolipids and 98.9% Dirhamnolipids (purified Di-RL). Treatment with Di-RL reduced inflammation and oxidative stress in UVB-irradiated hairless mouse skin. At a dose of 3 mg/kg, purified Di-RL reduced edema, epidermal thickening, leukocyte infiltration, myeloperoxidase activity, mast cell counts, antioxidant capacity depletion, lipid peroxidation, superoxide anion levels, and TNFα production. Collectively, these results underscore the therapeutic potential of Di-RL in the management of UVB-induced skin inflammation and oxidative stress.

## Graphical abstract

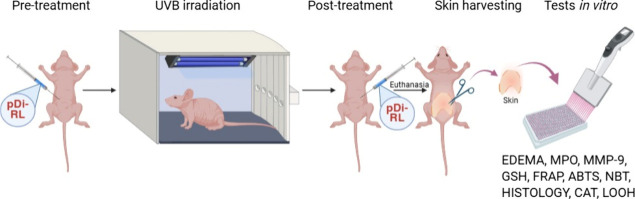

## Introduction

The skin is constantly exposed to sunlight, being the organ most susceptible to damage caused by ultraviolet radiation (UVR).^1^ Excessive and repeat exposures of skin to UVR promote an accelerated aging process, resembling chronological aging, which has been called photoaging.^2^ The skin’s photoaging process is linked to a persistent low-grade inflammation that increases the risk of carcinogenesis and metastasis.^2,3^ Besides to be the minor component of sunlight reaching Earth’s surface, ultraviolet B radiation (UVB) is the most effective light to induce skin cancer in animals.^4^ UVB causes DNA damage, mostly through cyclobutene pyrimidine dimer formation and photoproducts that induce epidermal-cell mutation, leading to the development of cancer cells.^4^ Excessive exposure to UVB also generates reactive oxygen species (ROS), such as hydrogen peroxide and superoxide anions, leading to DNA single strand breakage, changes in purine bases, lipid peroxidation in membranes, and oxidative protein modifications.^5^ In addition, UVB irradiation in skin cells stimulates immune system components, setting up an inflammatory response via generation of cytokines, including tumor necrosis factor (TNF) α, that will regulate the inflammatory response.^1,6^ The synergistic impact of ROS and inflammatory mediators on skin carcinogenesis has driven the search for compounds with antioxidant and anti-inflammatory properties to be applied in protecting skin photodamage.^3,6,7^

Rhamnolipids are glycolipid surfactants mostly produced by *Pseudomonas aeruginosa* as a mixture of congeners. They are composed of one or two molecules of rhamnose linked to β-d-(β-d-hydroxyalkanoyloxy) alkanoic acids (mono- and Di-RL congeners, respectively).^8,9^ Rhamnolipids are biodegradable, highly effective surface-active molecules that present low toxicity and an enormous market potential.^8,9^ Like other glycolipids, rhamnolipids exhibit a strong ability to destabilize cellular membranes by promoting the formation of ion channels and pores, which contributes to their effective activity against a wide range of bacteria, viruses, mycoplasma, and fungi.^9–12^ Beyond their well-established surface-active and antimicrobial functions, rhamnolipids have been reported to stimulate immune responses in plants and animals,^13–18^ promote wound healing,^12,19–23^ enhance growth performance and immune competence in broiler chickens,^24–27^ and exert antinociceptive effects in murine models.^28^ Given the established biological activities of rhamnolipids and the absence of studies addressing their role in UVB-induced skin injury, this study aimed to investigate the anti-inflammatory and antioxidant potential of Di-RL using a hairless mouse model of UVB-induced cutaneous inflammation and oxidative stress.

## Materials and Methods

### Production of Rhamnolipids

The microorganism used was the bacteria *Pseudomonas aeruginosa* PAO1 (ATCC 15692), stored in Luria–Bertani (LB) broth with glycerol (20% w/v) at −80 °C. Bacterial colonies grown on LB agar plates were preinoculated in Erlenmeyer flasks (125 mL capacity) containing LB medium (25 mL) and incubated in an orbital shaker. Subsequently, the preinoculum broth was inoculated (2% v/v) in 250 mL Erlenmeyer flasks containing 100 mL of salt medium (concentrations per liter: 3.0 g KH_2_PO_4_; 7.0 g K_2_HPO_4_; 0.2 g MgSO_4_.7H_2_O, 1.0 g (NH_4_)_2_SO_4_ and glycerol 3% v/v). All flasks were incubated at 37 °C and 200 rpm for 9 days.^8,28^ The cultures were interrupted by centrifugation (2500*g* for 10 min), and the supernatant was acidified at pH 2 by addition of 1 M HCl and stored at 4 °C for 5 days followed by 2500*g*/25 min/4 °C centrifugation. Rhamnolipid pellets were subjected to an extraction process with chloroform:methanol (9:1 ratio).^28^

### Purification of Dirhamnolipids

The purification of the crude extract of rhamnolipids was performed in a column packed with silica gel 60 PF254 (Merck) that had been previously activated with methanol. The method consists of two washing steps to remove nonpolar compounds and a third step with an extractor solvent. The presence of Di-RL rhamnoses and the absence of other carbohydrates were confirmed by thin-layer chromatography on silica (DC-Fertigfolien ALUGRAM Xtra SIL G/UV254, 20 × 20 cm) stained with orcinol.^28^

### Characterization and Purity of Dirhamnolipid

The rhamnolipid congeners were identified in a mass spectrometer (LCMS-8040, Shimadzu) by negative electrospray ionization (ESI). The position of the fatty acids of the isomeric pairs was obtained through the multiple reaction monitoring (MRM) mode at 20 V collision energy.^29,30^ The relative abundance of the congeners was obtained from the intensity of the peaks of the respective identified congeners, while the relative abundance of the isomers was determined by the intensity of the peaks of the key fragments produced by the removal of the terminal fatty acid.^31^ Purity was determined by nuclear magnetic resonance (Bruker Avance III 400 MHz) through spectral deconvolution. The degree of purity was calculated from the ratio between total hydrogen signal attributable to the analyte and the total hydrogen signal of the sample.^8^ NMR spectroscopy analysis also confirmed the absence of detectable lipopolysaccharide signals in the purified Di-RL fraction.

### Animals

The experiments were carried out with adult female hairless mice (HRS/J), with an average weight of 30 g. Mice were provided by the University Animal House and kept with free access to water and feed, controlled temperature (23 ± 2 °C), and light/dark cycle (12/12 h). All handling and care procedures were approved by the Research and Animal Welfare Ethics Committee of the State University of Londrina (certification number 10739.2019.32).

### Experimental and Irradiation Protocol

Hairless mice were randomly assigned to five experimental groups (*n* = 6 animals per group per experiment), and the results are representative of two independent experiments. Sample size was determined based on previous studies employing the same experimental model and end points,^6,7,32–35^ which consistently confirmed the minimum number of mice for the present experimental approach. The experimental groups were nonirradiated control (N-IC), irradiated control (IC), and irradiated animals treated with Di-RL at 0.3, 3.0, or 30.0 mg/kg (Figure 1A). All Di-RL dilutions were prepared in saline solution. The hairless mice were treated intraperitoneally 1 h before and 1 h after the end of exposure to UVB irradiation (Figure 1 A).^33^ The IC group was treated only with the vehicle (saline solution). The Di-RL doses tested were selected from the dose–response curve.

**Figure 1:**
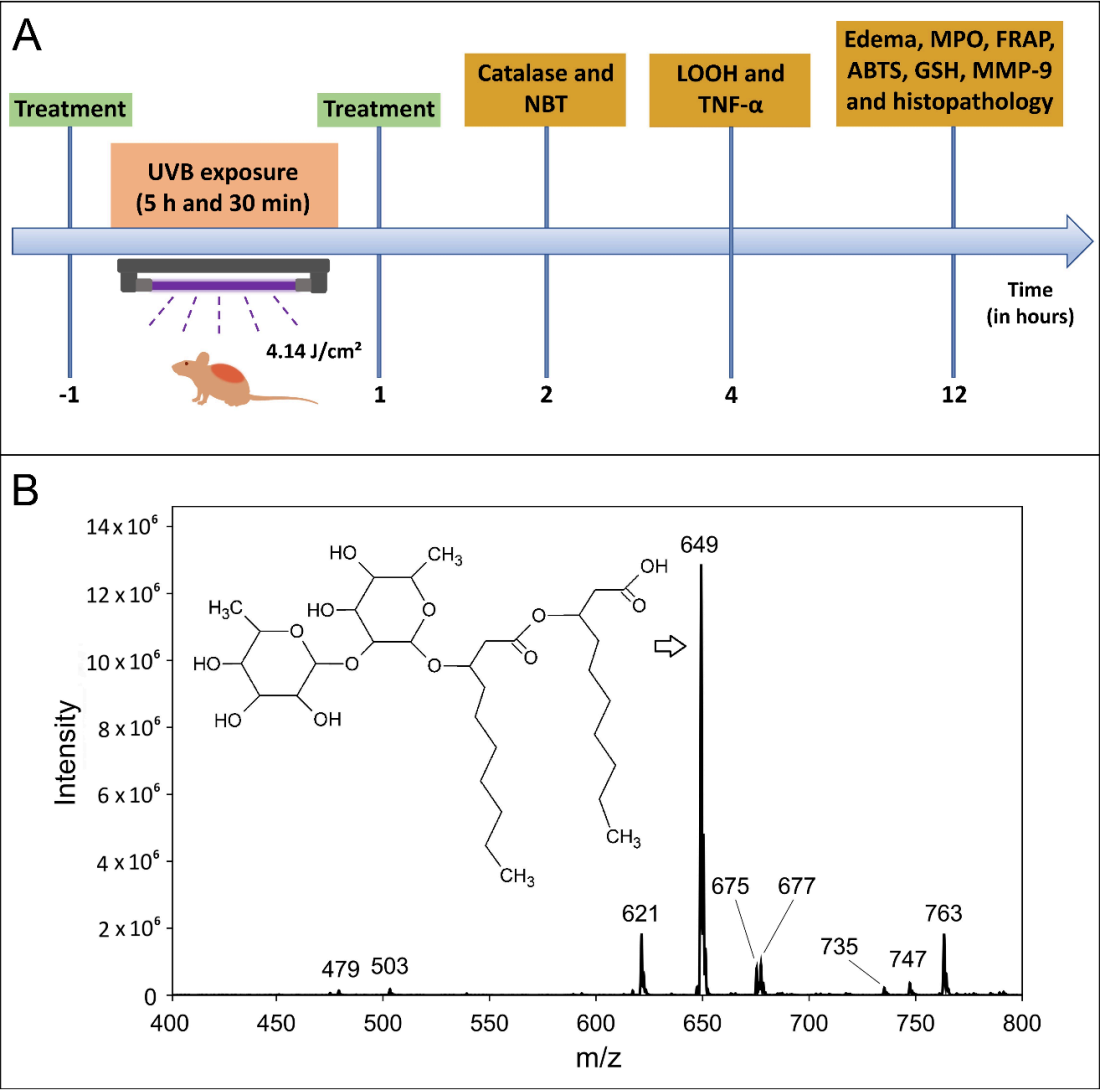
Schematic drawing of the *in vivo* UVB irradiation protocol (A). Inflammation and oxidative stress were induced at a dose of 4.14 J/cm^2^ (total exposure durations of 5 h and 30 min). The hairless mice were treated intraperitoneally 1 h before and 1 h after the end of UVB irradiation. Mice were euthanized 2 h after the end of irradiation for catalase and superoxide anion (NBT) assays, 4 h for lipid hydroperoxide (LOOH) and TNFα determination, and 12 h for skin edema, myeloperoxidase (MPO), ferric reducing antioxidant power (FRAP), ABTS radical scavenging, matrix metalloproteinase-9 (MMP-9), and histopathological analyses. In all cases, the dorsal skin was immediately collected. Most abundant Dirhamnolipid congener (Rha_2_C_10_C_10_) structure and mass spectra of pure Dirhamnolipids (B); the arrow indicates deprotonated Rha_2_C_10_C_10_.

All groups (except N-IC) were simultaneously exposed to 4.14 J/cm^2^ of UVB. The source of radiation was a fluorescent UVB lamp (Philips TL/12 RS 40 W) positioned 20 cm from the dorsal area of the mice.^32^ The intensity of the radiation was checked prior to the exposure with a radiometer (IL 1700, Newburyport, MA, USA). This dose of UVB was previously standardized by our group^33^ and represents an acute exposure to sunlight without protection^36^ that still limits the intensity of stimulation within the capability of the skin to allow observing induction and modulation by treatments.^33^

After the end of irradiation, mice were euthanized with 5% isoflurane and the dorsal skin was collected in 2 h (catalase activity and production of superoxide anion), 4 h (lipid hydroperoxides and TNFα dosage by ELISA), or 12 h (edema, myeloperoxidase activity, endogenous antioxidants, metalloproteinase-9 activity and histopathology). Edema was analyzed immediately after skin collection. The samples destined for histopathology were fixed in 4% paraformaldehyde, and the remaining samples were stored at −70 °C.^6^ The results obtained in the analysis of skin edema and myeloperoxidase activity were used to determine the optimal dose of Di-RL.

#### Skin Edema

Dorsal skin samples were collected with the aid of a 5 mm biopsy punch and were immediately weighed. The results were expressed in mg of skin.^6^

#### Myeloperoxidase (MPO) Activity

Neutrophil infiltration was verified by the activity of the enzyme MPO, which promotes the oxidation of *o*-dianisidine into a colored compound detected at 450 nm.^37^ The skin was crushed in 50 mM phosphate buffer (pH 6.0) with 0.5% hexadecyltrimethylammonium bromide (HTAB). The results were expressed as the number of neutrophils per milligram of skin.

#### FRAP and ABTS Assay

FRAP and ABTS assays were performed to evaluate the total antioxidant capacity. The samples were crushed in a 1.15% KCl solution. Absorbance readings were performed at 595 and 730 nm for FRAP and ABTS, respectively. The results were expressed as Trolox equivalents in nmol per milligram of skin.^38^

#### Reduced Glutathione (GSH) Assay

GSH levels were evaluated by colorimetric detection of 5-mercapto-2-nitrobenzoic acid at 405 nm, resulting from the breaking of the disulfide bond of 5,5′-dithiobis­(2-nitrobenzoic) acid (DTNB). The samples were crushed in a solution of 0.02 M EDTA and 50% trichloroacetic acid. The results were expressed as μM of GSH per mg of skin.^39^

#### Catalase Activity

The method is based on the drop in the concentration of hydrogen peroxide (H_2_O_2_) at 240 nm. The samples were crushed in a 0.02 M EDTA solution. The results were obtained by the difference between the initial (time 0) and final (30 s) readings and expressed in catalase units/mg of skin/min.^40^

#### Superoxide Anion (O_2_^•–^) Production

The method is based on the detection of formazan at 620 nm, resulting from the reduction of tetrazolium nitroblue (NBT) by O_2_^•–^. The samples were ground in 0.02 M EDTA. The results were expressed as optical density (OD) per 10 mg of skin.^34^

#### Lipid Hydroperoxide (LOOH) Production

The chemiluminescence test initiated by LOOH was used to evaluate the occurrence of oxidative stress.^41^ The skin samples were crushed in phosphate buffer (pH 7.4). The reading was performed on a BeckmanLS 6000SC β counter (Fullerton, CA, USA) in a noncoincident count (30 s) with a response range of 300 to 620 nm. The test was carried out at 30 °C for 120 min. The results were expressed as counts per minute (cpm) per milligram of skin.^7^

#### TNFα Production

TNFα production was quantitated by an ELISA kit according to the manufacturer's instructions (R&D systems).^6^ The skin samples were homogenized in saline (500 μL) with the aid of a tissue turrax (Biospec 985370) followed by a centrifugation step. TNFα production was quantitated in the supernatant by ELISA with readings at 490 nm and using the cytokine standard curve.

#### Matrix Metalloproteinase (MMP-9) Activity

MMP-9 activity was evaluated by zymography of polyacrylamide gel with sodium dodecyl sulfate (SDS) and gelatin. The samples were ground in a solution of Tris/HCl pH 7.4 with calcium chloride (CaCl_2_) and 1% proteinase inhibitors. The gels were incubated overnight (at 37 °C), stained bright blue, and decolorized in 20% acetic acid.^6^ The proteolytic activity of MMP-9 was analyzed through the intensity of the bands in the ImageJ (NIH) software.

#### Histopathological Analysis

Skin samples were fixed in 4% paraformaldehyde, embedded in paraffin, and sectioned with a thickness of 5 μm. Epidermal thickness (40× magnification) was evaluated with hematoxylin-eosin (H&E) staining by the Infinity Analyzer software (Lumenera R Software).^42^ Leukocyte infiltration was calculated per area using the ImageJ software (NIH).^6^ Mast cell infiltration in the dermis (40× magnification) was evaluated with Toluidine Blue staining by the Infinity Analyzer software (Lumenera R Software).^43^ Collagen fiber density (10× magnification) was evaluated with Masson’s trichrome staining using the ImageJ software (NIH).^44^ Histological analyses were performed in a blinded manner. For each animal, 3–6 histological sections were analyzed, and the measurements were averaged to obtain a single value per animal for statistical analysis.

### Statistical Analysis

Statistical analyses and graphical representations were performed using GraphPad Prism 7 (GraphPad Software Inc., San Diego, CA, USA). Data were analyzed by one-way ANOVA followed by Tukey’s post hoc test. The assumptions of normality and homogeneity of variances were assessed using the Shapiro–Wilk test and Brown–Forsythe test, respectively, and all data sets met these assumptions. A significance level of 5% (*p* < 0.05) was adopted for all statistical tests. Data are presented as mean ± standard error of the mean (SEM).

## Results

### Dirhamnolipid Production and Purification

Recently, we reported a simple, rapid, and cost-effective process for the production and purification of Di-RL from *Pseudomonas aeruginosa*.^28^ In the present study, we applied this procedure – consisting of two steps to obtain a crude rhamnolipid extract followed by a single-step purification^28^ – to yield a highly pure rhamnolipid mixture (99.5% purity (Table 1 and Figure S1)) containing 98.9% Di-RL, with Rha_2_C_10_C_10_ as the most abundant congener (Table 2, Figure 1B), hereafter referred to as pDi-RL.

**Table 1:** Rhamnolipid Production and Purification: Yields, Composition, and Purity

sample	rhamnolipid yield (g/L)	monorhamnolipid	**Dirhamnolipids** ^a^	**Rha**_**2**_ **C**_**10**_ **C**_**10**_ ^a^	**recovery rate** ^b^	**purity** ^c^
crude extract	1.7					
pDi-RL	0.9	1.1%	98.9%	76.7%	53.3%	99.5%

a Calculated through mass spectrometry (Table 2).

b Percentage of recovery of purified rhamnolipids from crude extract.

c Calculated through NMR proton spectral analysis (Figure S1).

**Table 2:** Chemical Composition and Relative Abundance of the Purified Rhamnolipids

structure	**[M–H]** ^–^	**MRM** ^a^	relative abundance (%)
Rha_1_C_10_C_10_	503		1.06
Rha_2_C_10_	479		0.81
Rha_2_C_8_C_10_ ^b^	621	451	6.88
Rha_2_C_10_C_8_ ^b^	621	479	4.11
Rha_2_C_10_C_10_	649		76.72
Rha_2_C_10_C_12:1_ ^c^	675	479	4.36
Rha_2_C_12:1_C_10_ ^c^	675	505	0.50
Rha_2_C_10_C_12_ ^d^	677	479	3.71
Rha_2_C_12_C_10_ ^d^	677	507	1.85

a Multiple reaction monitoring (Figure S2A–C).

b Isomers.

c Isomers.

d Isomers.

### pDi-RL Treatment Reduces Edema and Myeloperoxidase (MPO) Activity in UVB-Irradiated Skin of Hairless Mice

Edema and neutrophil recruitment are pathological events caused by a UVB-irradiation stimulus.^45^ pDi-RL treatment at 3.0 mg/kg significantly reduced skin edema induced by UVB irradiation (Figure 2A). No effect was found with higher and lower pDi-RL doses (i.e., 0.3 and 30 mg/kg) (Figure 2A); thus, it exhibited a bell-shaped effect. While the effects of rhamnolipids on edema have not yet been reported, evidence from studies investigating other glycolipids as therapeutic interventions^46,47^ provides indirect support for the present findings.

**Figure 2:**
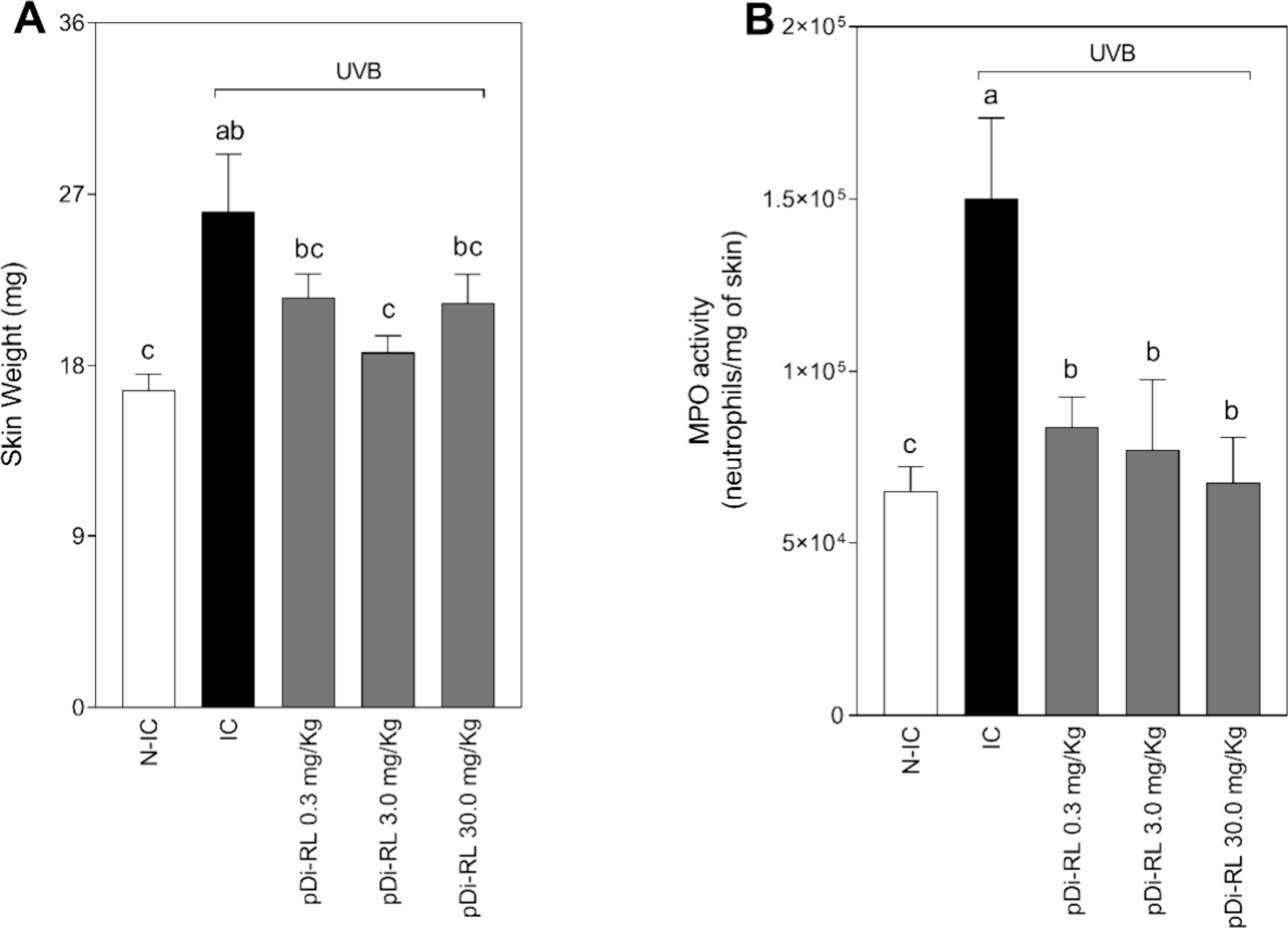
Dirhamnolipids reduce edema and myeloperoxidase (MPO) activity in UVB-irradiated skin of hairless mice. Mice were treated intraperitoneally with pDi-RL (0.3, 3.0, or 30.0 mg/kg) 1 h before and 1 h after the end of UVB irradiation. Skin edema (A) and MPO activity (B) were determined in samples collected 12 h after ending of irradiation. Bars show mean ± SEM (*n* = 6 mice/group/experiment) from two independent experiments. Different lowercase letters indicate statistically significant difference in one-way ANOVA followed by Tukey’s test *p*-value < 0.05. Nonirradiated control group (N-IC); irradiated control group (IC) (vehicle).

Neutrophil infiltration plays an important role in skin damage postirradiation.^7^ These cells enhance the inflammatory response through the production of a high amount of pro-inflammatory cytokines and reactive oxygen species.^48^ Myeloperoxidase (MPO) activity is commonly measured using a colorimetric assay that indirectly reflects neutrophil accumulation, as this enzyme is abundantly expressed in these cells.^37^ A significant increment of MPO activity was found in the UVB-irradiated group compared to the nonirradiated group (Figure 2B), and pDi-RL treatment significantly reduced the MPO activity with all tested doses (Figure 2B). Although no significant differences in MPO activity were observed among the tested doses, only the 3.0 mg/kg dose effectively attenuated skin edema (Figure 2A). As edema represents a functionally relevant inflammatory end point integrating vascular permeability and cellular responses,^49^ this dose was selected for subsequent experiments. As the MPO results suggested that pDi-RL treatment reduces skin inflammation by decreasing neutrophil recruitment and associated inflammatory events, we further investigated some of these possibilities.^48^

### pDi-RL Treatment Attenuates Skin Epidermal Thickening and Leukocyte Infiltration Induced by UVB Irradiation in Hairless Mice

UVB irradiation increases epidermal thickness^50^ and might be used as a quantitative parameter to assess skin inflammation^6^ (Figure 3A-D). Hematoxylin- and eosin-stained tissue sections showed significant dorsal skin epidermal thickening following UVB irradiation, with a 1.7-fold increase when the irradiated control group (30.10 ± 1.47 μm, Figure 3C) was compared to the nonirradiated control group (18.26 ± 0.74 μm, Figure 3B). This UVB-induced epidermal thickening was abrogated by treatment with pDi-RL at 3.0 mg/kg (17.20 ± 0.55 μm, Figure 3A and D). UVB irradiation also causes the recruitment of leukocytes including neutrophils.^33,34^ Indeed, the irradiated control group showed increased leukocyte infiltration, as quantified by using ImageJ software, compared with the nonirradiated control group (Figure 3E–G). Treatment with pDi-RL (3.0 mg/kg) markedly attenuated leukocyte infiltration (Figure 3H), in agreement with the MPO activity results (Figure 2B).

**Figure 3:**
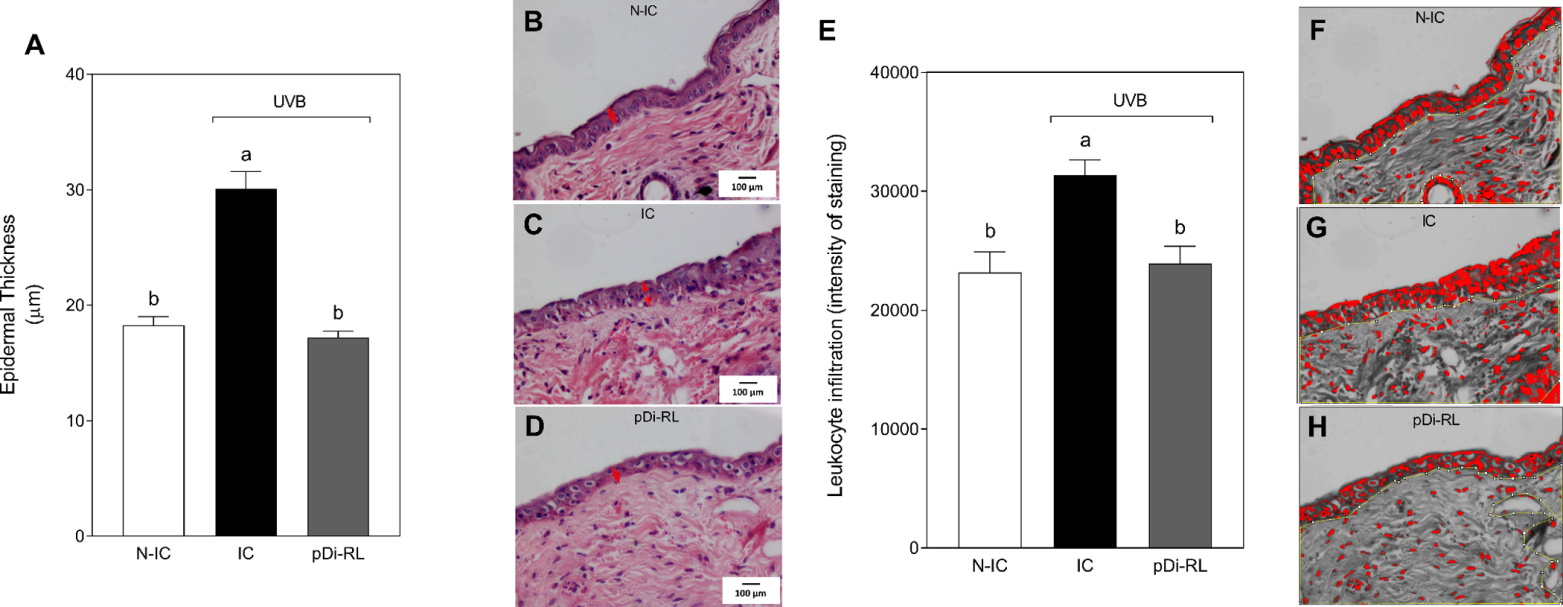
Dirhamnolipids reduce skin epidermal thickening induced by UVB irradiation. Mice were treated intraperitoneally with pDi-RL (3.0 mg/kg) 1 h before and 1 h after the end of UVB irradiation. Epidermal thickness (A) was evaluated using hematoxylin and eosin staining (H & E) in skin samples collected 12 h after the end of irradiation (B–D). Leukocyte infiltration (E) was evaluated using the ImageJ analyzes of the stained slices of skin samples collected 12 h after the end of irradiation (F–H). The sections stained with H & E were examined using light microscopy at 40× magnification. Bars show mean ± SEM (*n* = 6 mice/group/experiment) from two independent experiments. Different lowercase letters indicate statistically significant difference in one-way ANOVA followed by Tukey’s test *p*-value < 0.05. Nonirradiated control group (N-IC); irradiated control group (IC) (vehicle).

### pDi-RL Treatment Attenuates the UVB Irradiation-Induced Increase of Mast Cells

UVB irradiation significantly increases mast cell numbers in the skin, including their degranulation and release of reactive oxygen species.^51–53^ pDi-RL treatment attenuated the UVB irradiation-induced increase in mast cell counts in the skin of hairless mice (Figure 4A–D). There are no reports on the effects of rhamnolipids on mast cell counts in UVB irradiation models or in other inflammatory models.

**Figure 4:**
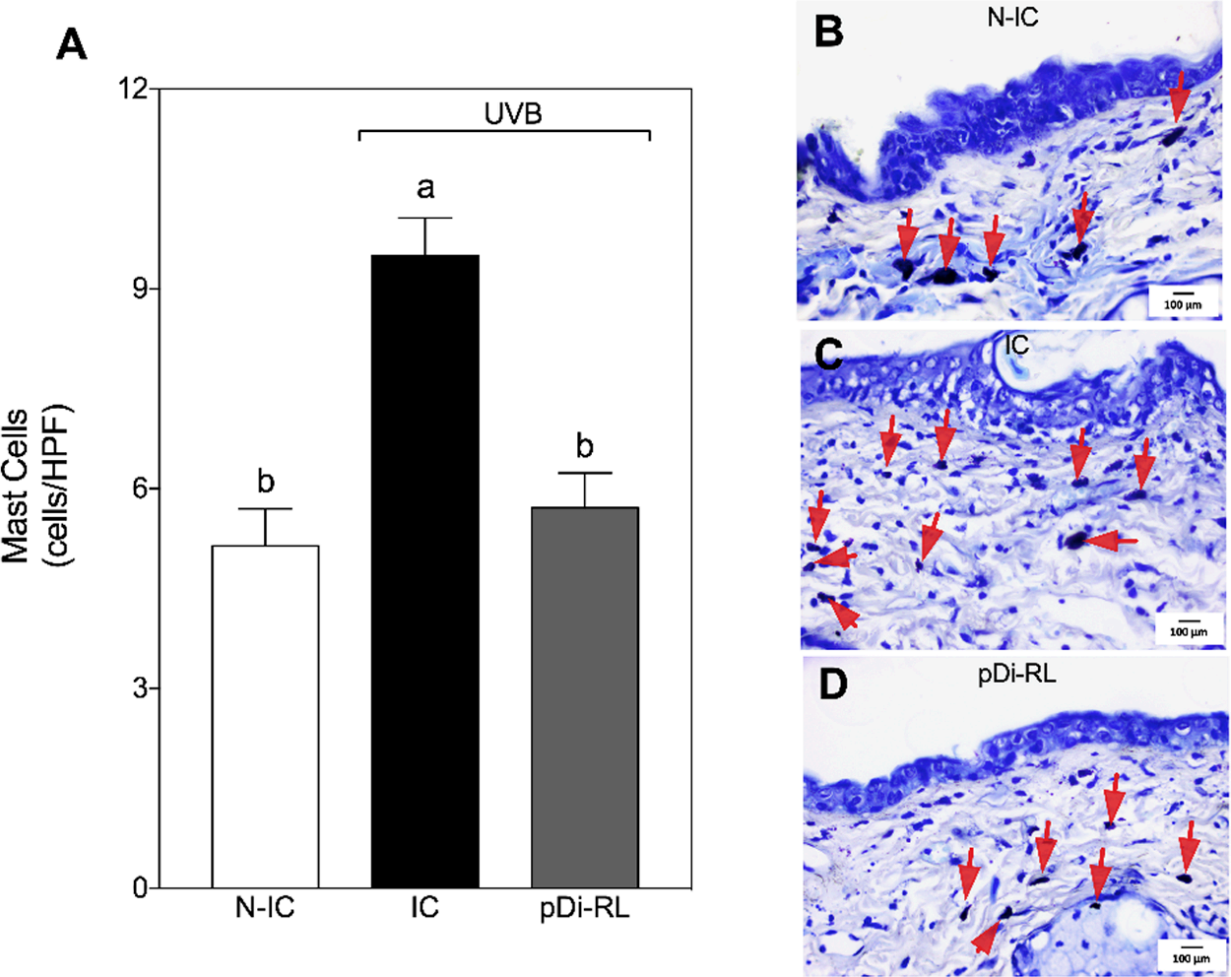
Dirhamnolipids reduce the UVB irradiation-induced increase of mast cells. Mice were treated intraperitoneally with pDi-RL (3.0 mg/kg) 1 h before and 1 h after the end of UVB irradiation. Mast cell counts were evaluated using blue toluidine in skin samples collected 12 h after the end of irradiation. The number of mast cells (A) in the sections stained with toluidine blue was examined using light microscopy at 40× magnification (B–D). Bars show mean ± SEM (*n* = 6 mice/group/experiment) from two independent experiments. Different lowercase letters indicate statistically significant difference in one-way ANOVA followed by Tukey’s test *p*-value < 0.05. Nonirradiated control group (N-IC); irradiated control group (IC) (vehicle).

### pDi-RL Treatment Reduces UVB Irradiation-Induced MMP-9 Activity and Damage to the Skin Collagen Fiber of Hairless Mice

MMP-9 is a gelatinase involved in skin collagen and components of the elastic fiber network degradation.^54^ Overexpression of MMP-9 in UVB-irradiated skin impacts development of skin cancer and accelerates photoaging.^55^ Impairment in the MMP-9 regulation might be associated with inflammatory skin diseases and invasive and metastatic cancer cells.^56^ Treatment with pDi-RL reduced the UVB irradiation-induced activity of MMP-9 (Figure 5A). In addition, histopathological analyses indicate that pDi-RL treatment presents a protection effect against skin collagen degradation, since blue-stained collagen fibers in the group treated with pDi-RL showed lower levels of damage in collagen fiber compared to the irradiated group (Figure 5B–E).

**Figure 5:**
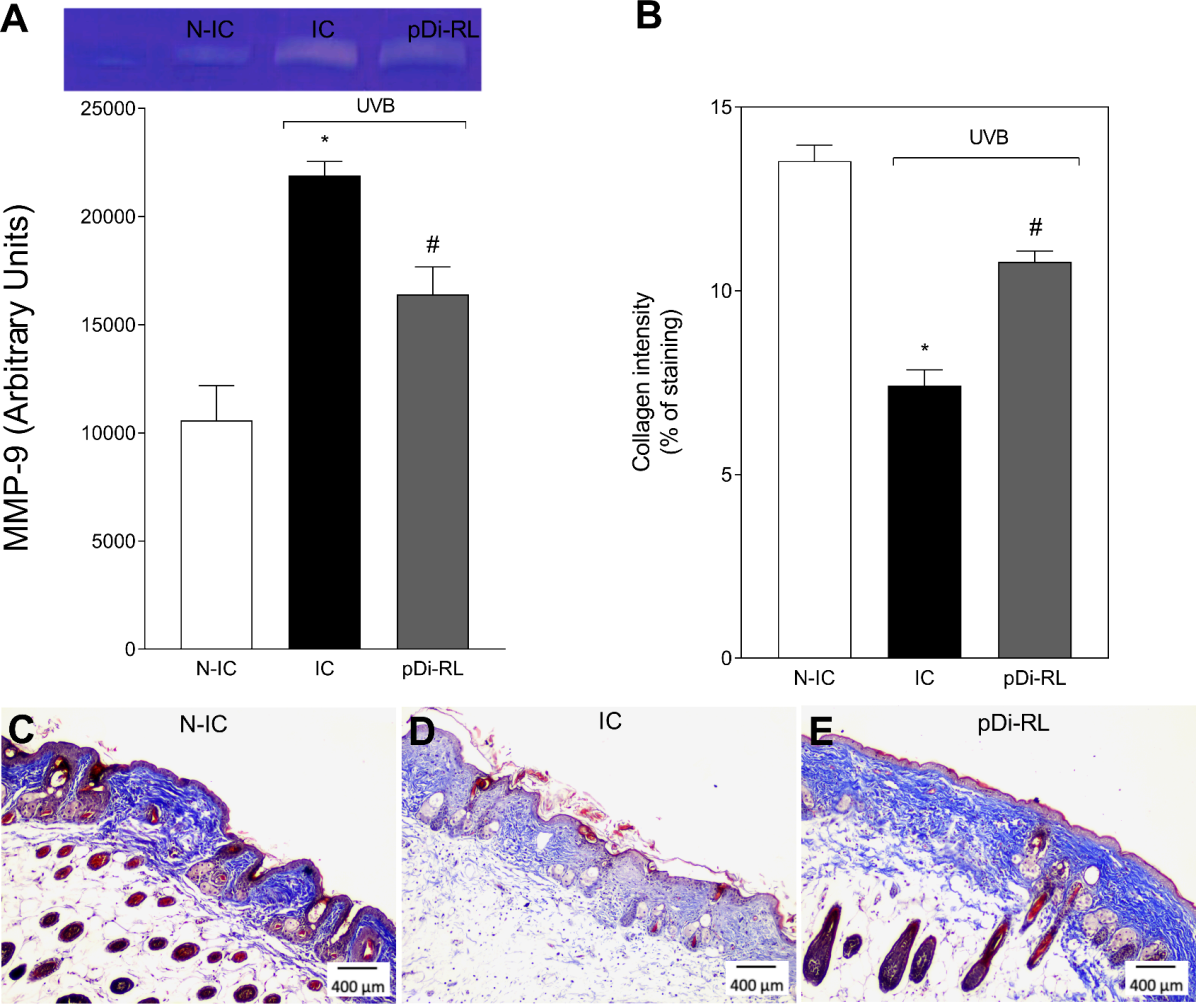
Dirhamnolipids reduce UVB irradiation-induced MMP-9 activity and damage to collagen fiber in skin. Mice were treated intraperitoneally with pDi-RL (3.0 mg/kg) 1 h before and 1 h after the end of UVB irradiation. The MMP-9 activity and collagen fiber formation were determined in samples collected 12 h after the end of irradiation. MMP-9 activity (A). Collagen fiber formation was evaluated using Masson’s trichrome staining (B–E). Collagen fiber intensity and bundles shown in blue were analyzed by the ImageJ program (10× magnification) (C–E). Bars show mean ± SEM (*n* = 6 mice/group/experiment) from two independent experiments. Different lowercase letters indicate statistically significant difference in one-way ANOVA followed by Tukey’s test *p*-value < 0.05. Nonirradiated control group (N-IC); irradiated control group (IC) (vehicle).

### pDi-RL Treatment Reduces the Depletion of the Antioxidant Capacity of Hairless Mice Skin Induced by UVB Irradiation

UVB irradiation, directly and indirectly (via the inflammatory response), promotes the production of ROS. To protect cells from ROS, mammalian tissues present numerous antioxidant systems, and balancing between both is crucial to preserve cellular functions. The overproduction of ROS and impairment of antioxidant defenses lead to oxidative stress.^6,54,57,58^ Both mast cells and neutrophils produce ROS.^52,53,59,60^ To evaluate the effect of pDi-RL treatment on skin antioxidant capacity, we performed the FRAP (ferric reducing antioxidant power) assay (Figure 6A) and the ABTS (2,2′-azinobis­(3-ethylbenzothiazoline-6-sulfonic acid)) radical scavenging assay (Figure 6B). UVB irradiation induced a decrease in FRAP values and ABTS radical scavenging capacity in the skin, confirming the pro-oxidant effect of UVB exposure.^6,7,35^ pDi-RL treatment reverted antioxidant depletion in both assays, preserving antioxidant capacity at levels comparable to the nonirradiated control (Figure 6A and B). To further evaluate the antioxidant effects of pDi-RL treatment, two key ROS-detoxifying systems were assessed, namely, reduced glutathione (GSH) levels and catalase (CAT) activity. UVB irradiation reduced significantly the GSH levels (Figure 6C) and CAT activity (Figure 6D).^6,7,35^ Following the same rationale of FRAP- and ABTS-assay results, pDi-RL treatment protected mouse skin against UVB-induced depletion of GSH and CAT (Figure 6C and D). In addition, the antioxidant effect of pDi-RL observed here may also be supported by the reduced infiltration of neutrophil and mast cells described above, suggesting a decrease in the production of pro-inflammatory and tissue-damaging ROS.^48,51,43^

**Figure 6:**
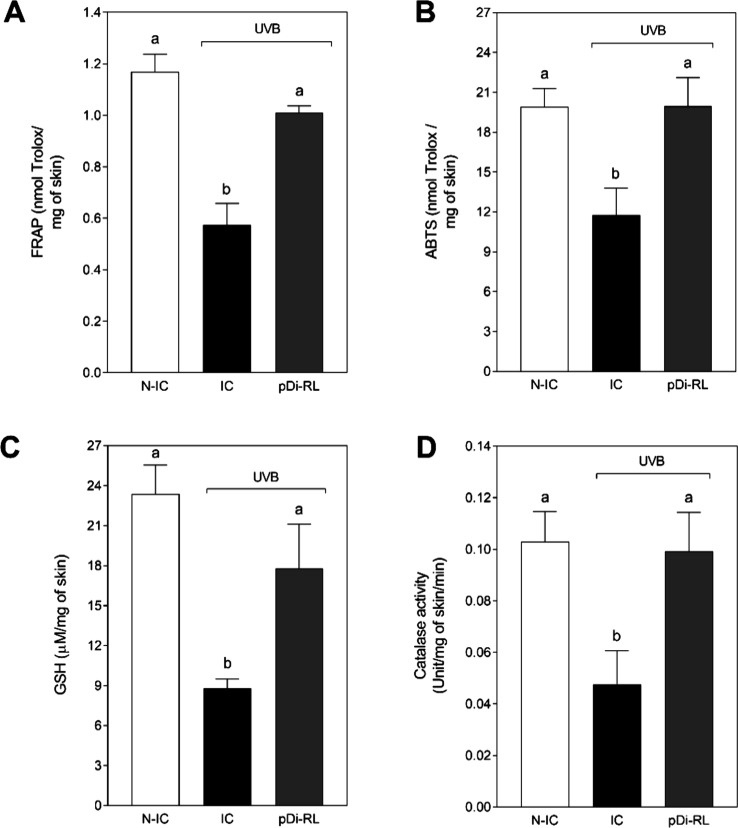
Dirhamnolipids reduce the depletion of antioxidant capacity of hairless mice skin induced by UVB irradiation. Mice were treated intraperitoneally with pDi-RL (3.0 mg/kg) 1 h before and 1 h after the end of UVB irradiation. The antioxidant capacity was determined by FRAP (A), ABTS (B), and GSH (C) assays in samples collected 12 h after the end of irradiation. The CAT activity (D) was determined in samples collected 2 h after the end of irradiation. Bars show mean ± SEM (*n* = 6 mice/group/experiment) from two independent experiments. Different lowercase letters indicate statistically significant difference in one-way ANOVA followed by Tukey’s test *p*-value < 0.05. Nonirradiated control group (N-IC); irradiated control group (IC) (vehicle).

### pDi-RL Treatment Attenuates Lipid Peroxidation (LPO) and Superoxide Anion (O_2_^•–^) Production in Hairless-Mice Skin Induced by UVB Irradiation

Skin fibroblasts exposed to UVB irradiation produce H_2_O_2_, leading to hydroxyl radical formation and consequent cellular damage, as well as LPO, a well-known harmful consequence of UV exposure.^7,58,61,62^ A *tert*-butyl hydroperoxide-initiated chemiluminescence (QL) assay was run to evaluate LPO in UVB-irradiated skin. A significative increase on LPO was observed in the irradiated control group when compared to the nonirradiated control group, which was completely reversed by pDi-RL treatment (Figure 7A). Upon UVB irradiation, skin keratinocytes convert molecular oxygen into a superoxide anion through the activity of nicotinamide adenine dinucleotide phosphate (NADPH) oxidase.^58,63^ A significative increase in O_2_^•–^ production was observed in the irradiated control group compared with the nonirradiated control group, which was completely reversed by pDi-RL treatment (Figure 7B). It is well established that UVB irradiation increases hydroperoxide and superoxide anion production in the skin of hairless mice.^6,7,35^ In the present study, pDi-RL treatment reversed these effects by reducing LOOH levels (Figure 7A) and superoxide anion production (Figure 7B).

**Figure 7:**
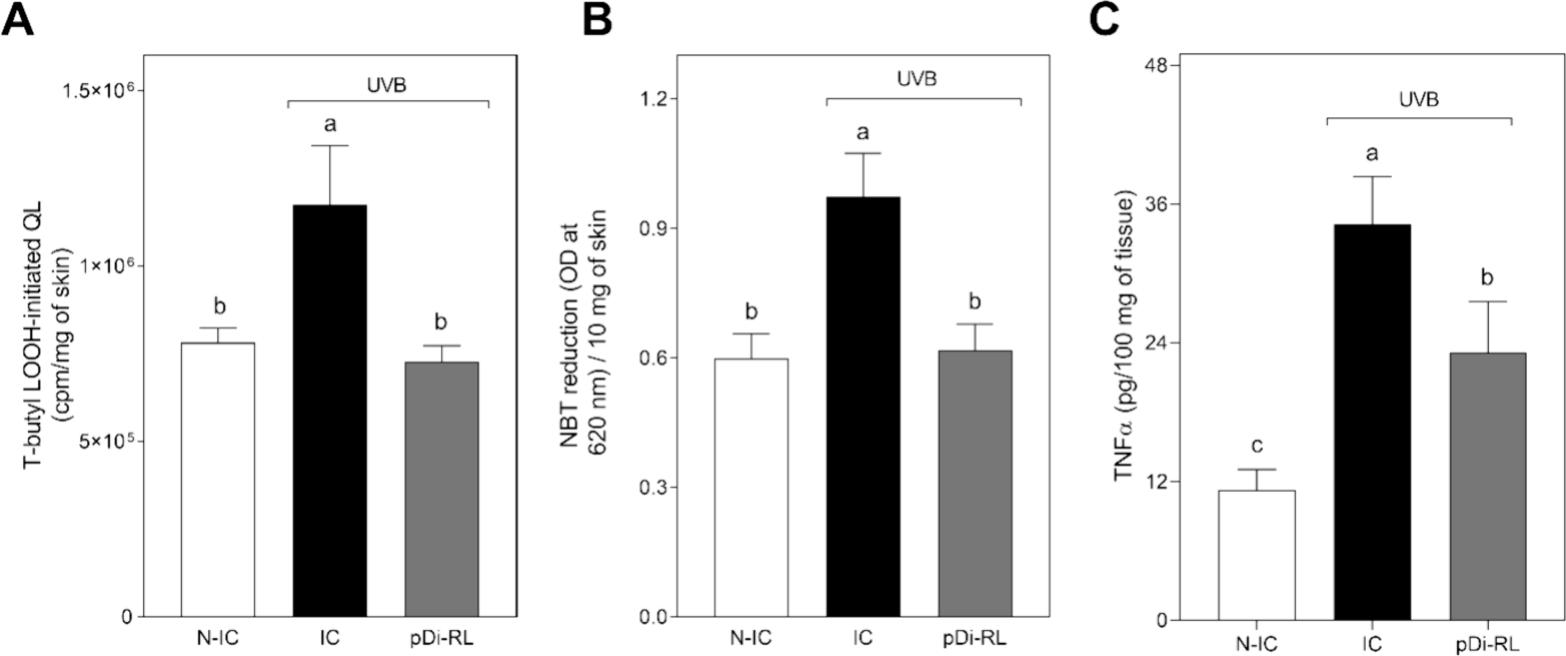
Dirhamnolipids prevent UVB irradiation-induced lipid peroxidation (LPO), superoxide anion (O_2_^•–^), and TNFα production in the skin of hairless mice. Mice were treated intraperitoneally with pDi-RL (3.0 mg/kg) 1 h before and 1 h after the end of UVB irradiation. Lipid peroxidation (A) was assessed by *t*-butyl lipid hydroperoxide (LOOH)-initiated chemiluminescence (CL) at 4 h, and superoxide anion production (B) was evaluated by a nitroblue tetrazolium (NBT) reduction assay at 2 h after the end of irradiation. TNFα levels (C) were determined by ELISA in samples collected 4 h after the end of irradiation. Bars show mean ± SEM (*n* = 6 mice/group/experiment) from two independent experiments. Different lowercase letters indicate statistically significant difference in one-way ANOVA followed by Tukey’s test *p*-value < 0.05. Nonirradiated control group (N-IC); irradiated control group (IC) (vehicle).

## Discussion

Rhamnolipids are secondary metabolites mainly produced by *Pseudomonas aeruginosa* that occur as heterogeneous mixtures of congeners.^64^ They are glycolipid biosurfactants composed of hydroxyalkanoyloxy alkanoic acids linked to one or two rhamnose units, giving rise to mono- and dirhamnolipid congeners, respectively.^8,9^ Owing to their well-established surfactant activity, rhamnolipids hold great potential for industrial and environmental applications.^64,65^ Here, we employed a previously described fermentation process to obtain a high proportion of Di-RL congeners (>95%), followed by a single-step purification procedure^28^ that yielded highly pure Di-RL (pDi-RL; 98.9% of Di-RL/99.5% of RL). In the present study, which applied a UVB-irradiated skin model in hairless mice, pDi-RL treatment reduced edema, MPO activity, leukocyte infiltration, and epidermal thickening and attenuated UVB-induced increases in mast cell counts, MMP-9 activity, and collagen fiber damage. Treatment with pDi-RL also reduced UVB-induced depletion of skin antioxidant capacity (e.g., FRAP, ABTS, GSH, and catalase), along with diminished superoxide anion production and LPO.

Di-RL have been reported to exhibit strong surface activity^64^ and to be more effective in applications such as stimulation of immune responses in animals and plants,^13–18^ targeted killing of myofibroblasts involved in skin scarring,^12,66^ and acceleration of wound healing.^21^ Topical treatment with an ointment containing 10 mg/mL Di-RL applied to full-thickness burn wounds in Sprague–Dawley rats resulted in accelerated wound closure.^21^ The authors suggested that Di-RL is effective in promoting normal wound healing and might overcome defects associated with impaired healing in chronic wounds.^21^ Clinical trials comparing Di-RL with conventional corticosteroid treatments showed that these compounds exhibit pronounced ameliorative effects on psoriasis, lichen ruber planus, neurodermatitis, and human wound healing.^67,68^ In addition, Di-RL reduced inflammation and nociceptive behaviors in mice in experimental models induced by carrageenan and formalin in the paw, as well as by acetic acid in the peritoneal cavity.^28^

Despite the clear importance of NF-κB and cytokines in inflammation, UVB skin pathology is initiated by the production of ROS. As an orchestrating group of molecules, ROS can induce varied activities. ROS can cause direct lesions to DNA, proteins, and lipids^5^ and initiate the production of other inflammatory molecules.^69^ In fact, ROS per se can induce tissue edema.^70,71^ ROS can induce the recruitment of neutrophils^59,60,69^ and mast cell degranulation.^52,53^ Mast cell activation further contributes to neutrophil recruitment.^32,72^ Neutrophils represent an essential leukocyte both in protecting from infection and causing tissue damage, and both activities are dependent on their production of ROS.^73–75^ In fact, neutrophils also activate MMP.^76,77^ Therefore, ROS are upstream and initiating molecules of the skin pathology observed upon UVB irradiation. In line with this, prior evidence demonstrated that subcutaneous treatment with Di-RL (3 mg/kg) inhibited leukocyte migration and superoxide anion production in mice with carrageenan-induced cutaneous inflammation and peritonitis.^28^ Accordingly, in the present study, the effects observed with pDi-RL are consistent with the inhibition of UVB-induced damage through targeting oxidative stress. Treatment with pDi-RL reduced the levels of superoxide anion and lipid peroxidation. From a different perspective, pDi-RL enhanced endogenous antioxidant defenses, as observed by increased FRAP and ABTS activities, which detect a general rise in diverse antioxidants that act through iron reduction and ROS scavenging. It was also observed that pDi-RL treatment increased the levels of GSH, an endogenous antioxidant as well as catalase, an essential enzyme in the degradation of hydrogen peroxide.^78–81^ These results are consistent with the inhibition of inflammation (e.g., skin edema, MPO activity, mast cell counts, and TNFα production)^6,82^ and tissue damage (e.g., MMP-9 zymography and collagen density staining) induced by UVB^83,84^ in animals treated with pDi-RL.

ROS also induces the production of cytokines such as TNFα, which is in turn part of the mechanisms orchestrating inflammation in varied conditions including UVB irradiation inflammation. TNFα is an important cytokine in neutrophil recruitment^85,86^ and activation of NADPH oxidase^87,88^ as demonstrated in previous studies. Using a model of inflammation triggered by KO_2_, a donor of superoxide anion, evidence supports that this ROS induces TNFα production. In turn, TNFα mediates the inflammatory and oxidative stress triggered by the superoxide anion, because pain, the increase of MPO activity, and oxidative stress were amenable by treatment with etanercept (a soluble TNFR2 receptor) or TNFR1 deficiency.^69^ Nevertheless, there is also a feedback upregulation loop since TNFα injection causes pain and MPO activity increase that were reduced by a NADPH oxidase inhibitor (apocynin) and a SOD mimetic (TEMPOL).^69^ Indeed, TNFα mediates a UVB irradiation inflammatory effect as observed by different genetic and pharmacological approaches.^89–91^ Another feedback upregulation loop involved TNFα induction of MMP-9, in which MMP-9 maturates pro-TNFα into active TNFα.^92^ Thus, it is possible to infer that the pDi-RL inhibition of TNFα production adds to explain its anti-inflammatory and antioxidant effects in UVB-irradiated skin.

Although it is a different condition since it is the treatment when a large skin damage has already been caused, the effects of rhamnolipids on wound healing are also in line with the present findings. Prior evidence demonstrates that rhamnolipids accelerate wound healing through several mechanisms (e.g., keratinocyte stimulation, granulation tissue formation, fibroblast proliferation, and collagenesis) in both cellular and animal models.^19–23^ Rhamnolipids enhanced the rate of wound contraction and improved collagenesis, reinforced by higher DNA, total protein, and hexosamine content of the regenerated tissue at the wound site.^20^ Rhamnolipids also showed activity against *Staphylococcus aureus* ATCC6588, suggesting that protecting the wound site from bacteriological infection could be an additional mechanism.^20^ Rhamnolipid extract accelerated wound healing in BALB/c mice excisional wound^22^ by mechanisms involving enhanced phosphorylation of Smad3 in fibroblasts and increased levels of proteins TGF-β 1 and α-SMA at the wound site. Thus, this previous study suggested that a rhamnolipid extract acceleration of wound healing involves the TGF-β 1 signaling pathway.^22^

We describe a sequence of experiments showing that pDi-RL treatment consistently reduces inflammation and oxidative stress in UVB-irradiated skin. As we mentioned earlier, there is no previous report on rhamnolipid effects on UVB skin pathology as we have reported here. Moreover, the reported biological and biomedical properties of rhamnolipids, along with those of other microbial glycolipids, provide additional support for the present findings. For instance, treatment with nebulized sophorolipid has decreased edema compared to vehicle-treated asthmatic mice challenged with ovalbumin,^46^ and topical application of glycolipids (monogalactosylmonoacylglyceride­(2*S*)-1-*O*-[(6*Z*,9*Z*,12*Z*,15*Z*)-octadeca-6,9,12,15-tetraenoyl]-3-*O*-β-d-galactopyranosylglycerol) from the microalgae *Isochrysis galbana* reduces edema on skin inflammation of murine in a 12-*O*-tetradecanoylphorbol-13-acetate (TPA) induced model, which also depends on ROS production.^47^ The glycolipid surfactant 4-O-[(4′,6′-di-*O*-acethyl-2′,3′-di-*O*-alkanoyl)-β-d-mannopyranosyl] *meso*-erythritol (MEL) produced by the yeast *Pseudozyma antarctica* inhibited secretion of inflammatory mediators from the mast cells.^93^ Low molecular weight fucoidans (complex sulfated polysaccharide isolated from marine brown algae) attenuated UVB-induced damage in human foreskin fibroblast (HS68) cells^94^ and in HR-1 hairless mice^95^ by inhibiting UVB stimulation of gelatinases MMP-9.^94,95^ Previous reports have indicated that rhamnolipids exhibit low or even negligible direct *in vitro* antioxidant activity in assays such as FRAP and ABTS.^96,97^ This lack of rhamnolipid activity in FRAP and ABTS assays is reasonable because of their chemical structure and limited ability to donate electrons or hydrogen atoms to neutralize free radicals. In contrast, rhamnolipids have been shown to mitigate oxidative stress in plant models.^9,98^ For example, their application enhanced resistance to *Alternaria alternata* infection in cherry tomatoes by increasing the activity of antioxidant enzymes, including catalase and superoxide dismutase, and elevating glutathione levels, ultimately reducing excessive ROS accumulation.^98^ Similarly, dietary supplementation with rhamnolipids improved antioxidant status in broiler chickens by increasing total antioxidant capacity as well as glutathione peroxidase and superoxide dismutase activities during growth.^99^ Together, these findings^98–100^ suggest that the antioxidant effects of pDi-RL observed in the present study are more likely associated with the stimulation of endogenous antioxidant defenses rather than with direct radical-scavenging activity. However, as compound-only controls were not performed, potential assay interference due to the surfactant nature of rhamnolipids cannot be entirely excluded.

In the present study, pDi-RL treatment attenuated several harmful effects of UVB exposure, including edema formation, epidermal thickening, collagen fiber damage, increased myeloperoxidase and MMP-9 activities, reduced antioxidant capacity, elevated mast cell counts, lipid peroxidation, and increased TNFα and superoxide anion production.

It is noteworthy that previous studies have reported that subcutaneous administration of Di-RL in Swiss–Webster mice induces low toxicity at doses as high as 120 mg/kg per day.^21^ Thus, it is reasonable to infer that the substantially lower dose employed in the present study (3 mg/kg) is not expected to result in systemic toxic effects. Supporting this interpretation, in a different experimental setting, treatment with 3 mg/kg Di-RL did not affect the animals’ responsiveness to mechanical stimulation assessed by an electronic aesthesiometer, indicating the preservation of normal sensory function.^28^ In addition, another report demonstrates that rhamnolipids have a favorable safety profile following oral administration, with reported ED_50_ values greater than 5000 mg/kg.^100^ Although these observations suggest an acceptable safety profile under the experimental conditions tested, further studies including comprehensive systemic toxicity evaluation would be valuable to strengthen the safety assessment. Moreover, the intraperitoneal administration used in the present work may be regarded as an initial approach to demonstrate biological activity *in vivo*, while future investigations exploring topical delivery strategies could contribute to advancing the translational relevance of these findings.

Finally, it is important to state some limitations of our study. Although we have discussed the mechanisms of disease triggered by UVB with the aim of adding the context in which the present data fits in, we have not assessed all potential markers altered in this model. Our data was limited to the following parameters: skin edema, MPO activity, leukocyte infiltration and epidermal thickening in H&E stained slices, mast cell counts in toluidine blue stained slices, MMP-9 activity, collagen fiber damage in Massons tricrome stained slices, GSH levels, FRAP, ABTS, and catalase activities, lipoperoxidation, and O_2_^•–^ and TNFα production. Additional potential targets would give further mechanistic insight into the pDi-RL activity; therefore, follow-up studies further addressing pDi-RL mechanisms of action would be valuable. For instance, intracellular signaling pathways (e.g., NFκB), enzymes (e.g., NADPH oxidase), other cytokines (e.g., IL-1β), DNA damage, and apoptosis were not assessed herein. The present UVB model mimics acute, unprotected sun exposure, as may occur during recreational activities or occupational outdoor work without adequate photoprotection.^36^ Investigating the activity of pDi-RL under chronic and repetitive UVB exposure would also be important to have better insight on its prolonged applicability and activity against chronic modifications caused by UVB.

## Conclusions

Rhamnolipids are natural, biodegradable, and low-toxicity surfactants with high surface activity that are part of the rapidly expanding global market for green amphiphilic molecules.^101^ They present great biomedical potential, ranging from antimicrobial, immunopotentiators, to wound healing accelerators.^12^ We reported that treatment with pDi-RL reduces inflammation and oxidative stress in UVB-irradiated skin. These results provide a basis for future investigations into the therapeutic potential of pDi-RL, particularly their antioxidant and anti-inflammatory effects in protecting the skin against photodamage. Further attention should be dedicated not only to evaluating the molecular mechanisms underlying the therapeutic effects of pDi-RL but also to two additional topics: (i) the development of formulations containing pDi-RL and (ii) the investigation of potential synergism between their main reported activities – antimicrobial,^11^ wound healing,^20^ and analgesic properties,^28^ with the antioxidant and anti-inflammatory activities, here described. Taken together, these activities indicate that pDi-RL might be a unique therapeutic approach with antimicrobial, wound healing, analgesic, antioxidant, and anti-inflammatory properties useful to skin pathologies, including those triggered by UVB as described herein.

## Supplementary Material



## Data Availability

The data presented in this study are available from the corresponding author upon reasonable request. No additional registration or access requirements apply.

## Notes

Relative purity analysis of purified Dirhamnolipids (pDi-RL) by proton NMR quantification; Mass spectra of the key fragment peaks of the isomers obtained after removal of the terminal fatty acid (PDF)
