# Supplementary material for: Dirhamnolipids from Pseudomonas aeruginosa PAO1 Protect Hairless Mouse Skin from UVB-Induced Inflammation and Oxidative Stress
Source: ACS Omega. 2026 Jun 19;11(25):36487–500. doi: 10.1021/acsomega.5c09252 (PMC13325137; doi:10.1021/acsomega.5c09252)
Supplement: Supplementary file 1 [file ao5c09252_si_001.pdf]

Supplementary Material

Di-rhamnolipids from *Pseudomonas aeruginosa* PAO1 Protect Hairless Mouse Skin from UVB-Induced Inflammation and Oxidative Stress

Isadora Caroline Sawoniuk[1], Ingrid Caroline Pinto[2], Priscila Saito[2], Jonathan Ratko[1], Kamila B. B. Wessel[1], Renata M. Martinez[2], Cesar A. Tischer[1], Ricardo Luís Nascimento de Matos[2], Thaísa Maria da Roda Lino[1], Nicole Caldas Pan[1], Laura de Oliveira Semeão[2], Ana Paula Frederico R. L. Bracarense[3], Waldiceu A. Verri[4], Rubia Casagrande[2*], Josiane A. Vignoli[1], Doumit Camilios-Neto[1*]

[1]Departamento de Bioquímica e Biotecnologia, Centro de Ciências Exatas, Universidade Estadual de Londrina, 86057-970, Londrina, Brazil.

[2]Departamento de Ciências Farmacêuticas, Centro de Ciências da Saúde, Universidade Estadual de Londrina, , 86038-440, Londrina, Brazil.

[3]Departamento de Medicina Veterinária, Centro de Ciências Agrárias, Universidade Estadual de Londrina, 86057-970, Londrina, Brazil.

[4]Departamento de Imunologia, Parasitologia e Patologia Geral, Centro de Ciências Biológicas, Universidade Estadual de Londrina, 86057-970, Londrina, Brazil.

*Correspondence: camiliosneto@uel.br and rubiacasa@uel.br

Nuclear magnetic resonance (NMR) Determination of Rhamnolipids Purity

All signals were calibrated according to the $(CD_3)_2SO$ solvent at 2.50 ppm. Manual phase correction, signal-to-noise ratio adjustment, and baseline correction were applied for accurate signal determination. Subsequently, deconvolution was applied with manual Gaussian adjustment of the area of all signals to minimize adjustment errors [1]. The purity was estimated from the ratio between the total hydrogen mass of the rhamnolipids and the total hydrogen mass of the sample [2]. The $(CD_3)_2SO$ signal was disregarded in the analysis. All spectral processing was performed using TopSpin 4.5.0 (Bruker, Germany).

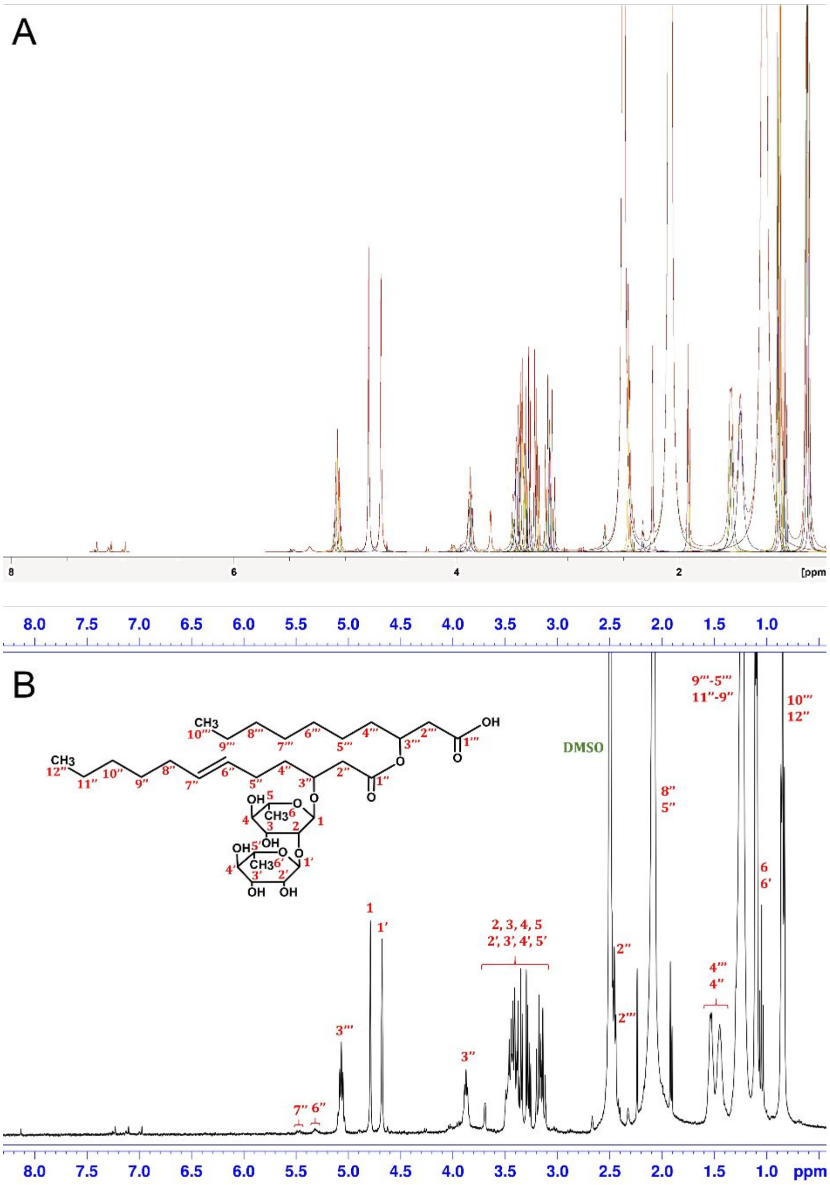

Figure S1 Relative purity analysis of purified Di-rhamnolipids by nuclear magnetic resonance proton-quantification. Deconvolution sum of each hydrogen signal present in the rhamnolipids nuclear magnetic resonance proton spectra (A). Calculated area for each signal from the deconvolution of the hydrogen signals in rhamnolipid spectrum, with the integrated regions and values (B).

Fatty acid positions within the isomeric rhamnolipid pairs were assigned by MRM analysis

Rhamnolipid congeners were identified by negative electrospray ionization (ESI) using a mass spectrometer (LCMS-8040, Shimadzu). The positions of the fatty acids in the isomeric pairs were determined through multiple reaction monitoring (MRM) mode at a collision energy of 20 V [3,4]. The relative abundance of the isomers was determined by the intensity of the peaks of the key fragments produced by the removal of the terminal fatty acid [5] (Figure S2).

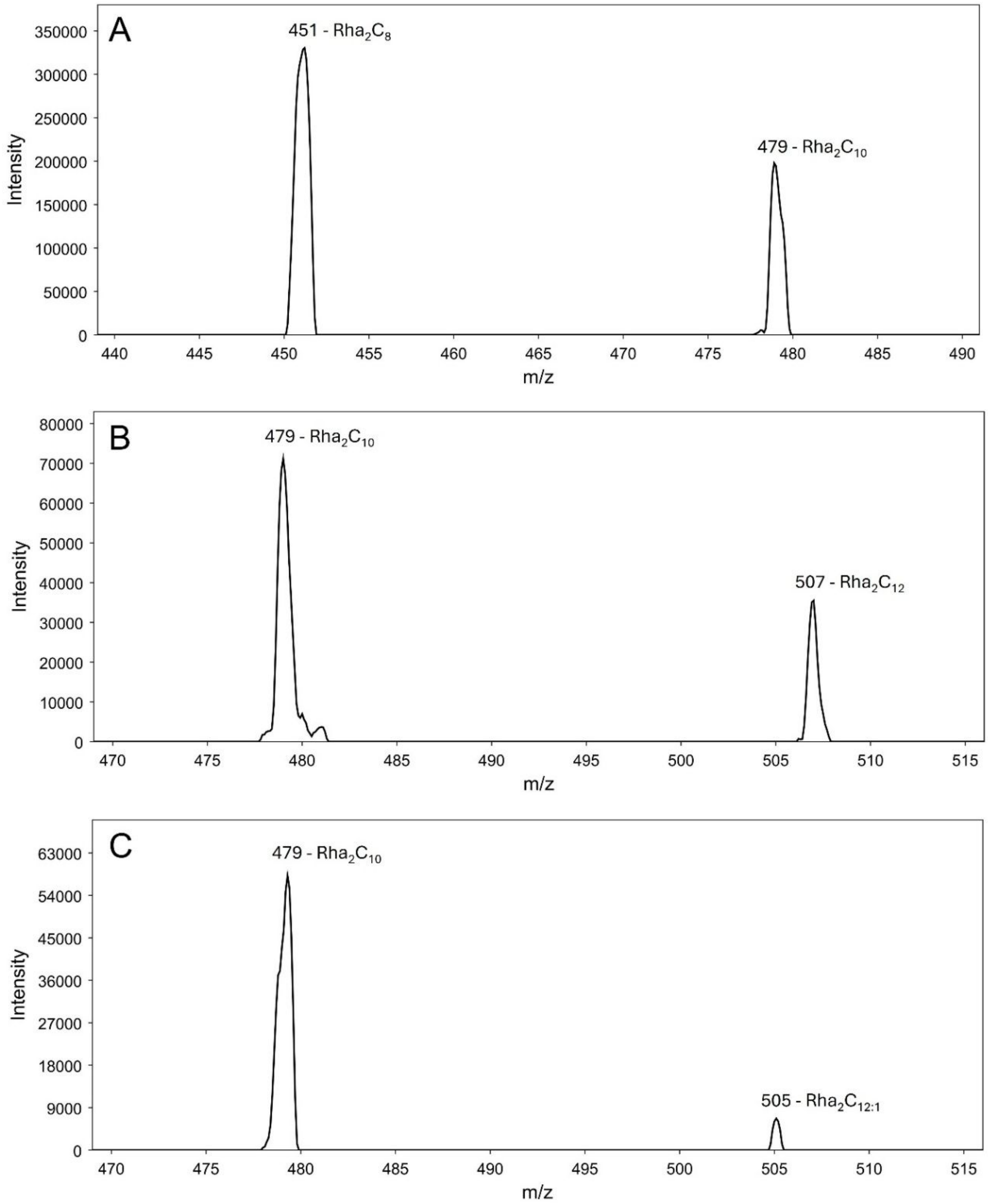

Figure S2 Mass spectra of the key fragment peaks of the isomers after removal of the terminal fatty acid. Isomers $Rha_2C_8C_{10}$/$Rha_2C_{10}C_8$ (A). Isomers $Rha_2C_{10}C_{12}$/$Rha_2C_{12}C_{10}$ (B). Isomers $Rha_2C_{10}C_{12:1}$/$Rha_2C_{12:1}C_{10}$ (C).

Original gels of zymography: MMP-9 activity

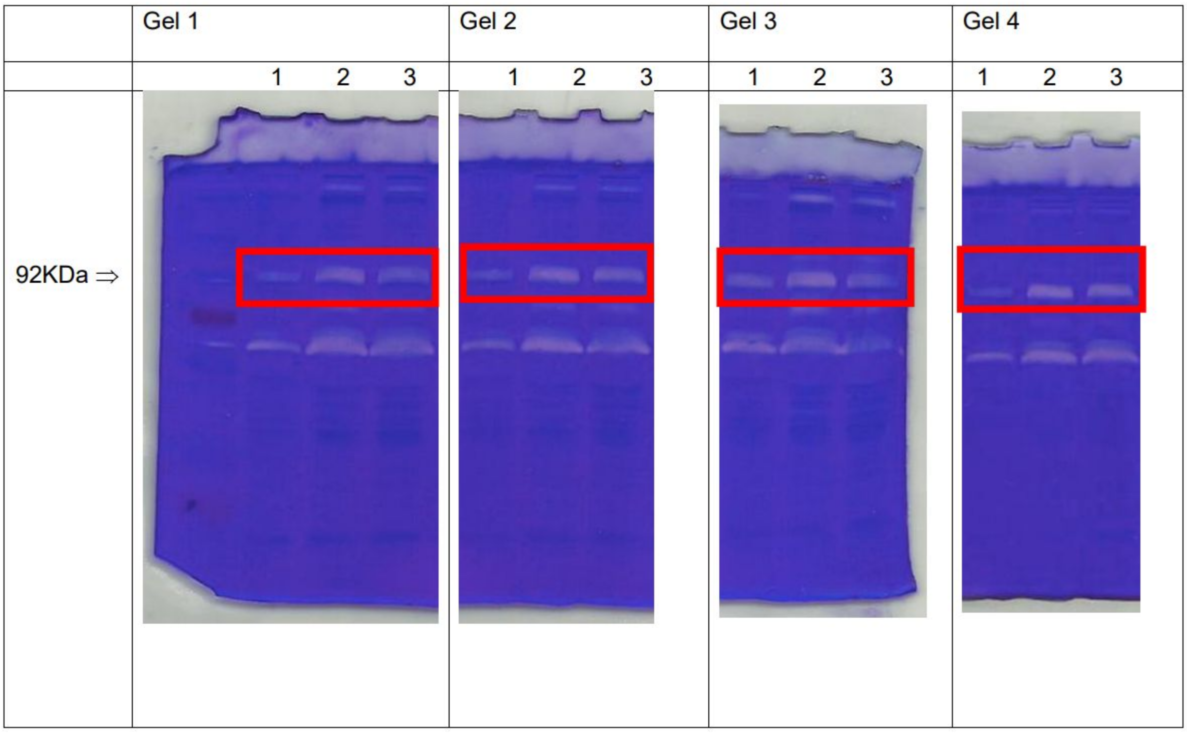

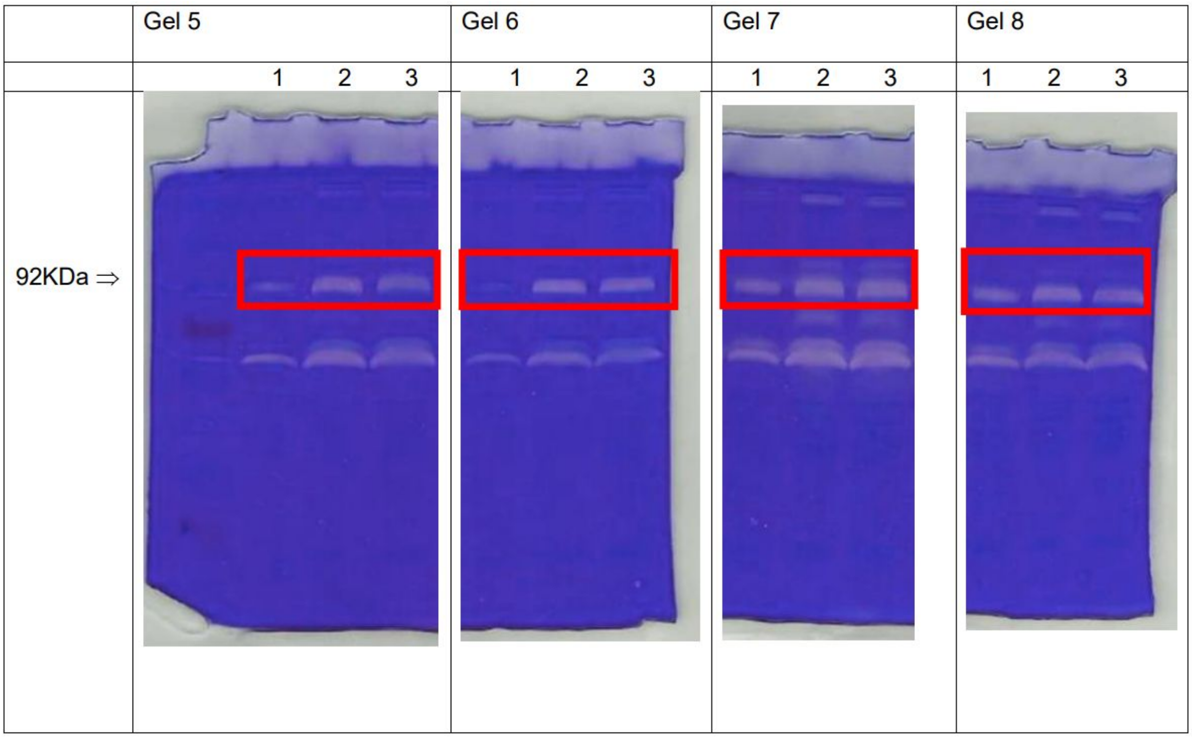

Figure S3 Original gels of zymography: MMP-9 activity. The sequence of groups in each gel is the same. Band 1: non-irradiated control group. Band 2: UVB irradiation + vehicle group. Band 3: UVB irradiation + di-rhamnolipids (3.0 mg/Kg). MMP-9 (92 Kda) bands are indicated with red rectangles. Each gel used a pool sample of 2 mice per group. There are 8 gels, thus, 16 mice were used per group. There were 3 groups, thus, a total of 48 mice were used.

References

(1) Fernández-Cruz, E.; de la O, V.; Fernández-Diaz, C. M.; Matía-Martín, P.; Rubio-Herrera, M. Á.; Amigó, N.; Calle-Pascual, A. L.; Martínez, J. A. Urinary Hippuric Acid as a Sex-Dependent Biomarker for Fruit and Nut Intake Raised from the EAT-Lancet Index and Nuclear Magnetic Resonance Analysis. *Metabolites* **2025**, *15* (6), 348. https://doi.org/10.3390/metabo15060348.

(2) Wessel, K. B. B.; Mello, A. P.; Amador, I. R.; Manchope, M. F.; Morelli, N. R.; Franciosi, A.; Zaninelli, T. H.; Bertozzi, M. M.; Tischer, C. A.; Martinez, R. M.; Pan, N. C.; Baracat, M. M.; Casagrande, R.; Verri, W. A.; Camilios-Neto, D.; Vignoli, J. A. Targeted-Produced Dirhamnolipids from *Pseudomonas Aeruginosa* Induce Antinociception in Mice. *ACS Omega* **2025**. https://doi.org/10.1021/acsomega.5c03648.

(3) Bahia, F. M.; de Almeida, G. C.; de Andrade, L. P.; Campos, C. G.; Queiroz, L. R.; da Silva, R. L. V.; Abdelnur, P. V.; Corrêa, J. R.; Bettiga, M.; Parachin, N. S. Rhamnolipids Production from Sucrose by Engineered Saccharomyces Cerevisiae. *Sci Rep* **2018**, *8* (1), 2905. https://doi.org/10.1038/s41598-018-21230-2.

(4) Rudden, M.; Tsauosi, K.; Marchant, R.; Banat, I. M.; Smyth, T. J. Development and Validation of an Ultra-Performance Liquid Chromatography Tandem Mass Spectrometry (UPLC-MS/MS) Method for the Quantitative Determination of Rhamnolipid Congeners. *Appl Microbiol Biotechnol* **2015**, *99* (21), 9177–9187. https://doi.org/10.1007/s00253-015-6837-1.

(5) Monteiro, S. A.; Sassaki, G. L.; de Souza, L. M.; Meira, J. A.; de Araújo, J. M.; Mitchell, D. A.; Ramos, L. P.; Krieger, N. Molecular and Structural Characterization of the Biosurfactant Produced by Pseudomonas Aeruginosa DAUPE 614. *Chem Phys Lipids* **2007**, *147* (1), 1–13. https://doi.org/10.1016/j.chemphyslip.2007.02.001.
